# Poly[[hexa­aqua­bis­(μ_3_-pyrimidine-4,6-dicarboxyl­ato)dicalcium] dihydrate]

**DOI:** 10.1107/S160053681204617X

**Published:** 2012-11-14

**Authors:** Wojciech Starosta, Janusz Leciejewicz

**Affiliations:** a Institute of Nuclear Chemistry and Technology, ul.Dorodna 16, 03-195 Warszawa, Poland

## Abstract

The polymeric structure of the title compound, {[Ca_2_(C_6_H_2_N_2_O_4_)_2_(H_2_O)_6_]·2H_2_O}_*n*_, is built up of mol­ecular layers composed of Ca^II^ ions bridged by both ligand N and O atoms with one of the O atoms being bis-monodentate. Two adjacent Ca^II^ ions are bridged by these O atoms, forming a centrosymmetric dimer which is the building unit of the structure. The dimers are nodes of a cross-linked mol­ecular layer parallel to (101). The Ca^II^ ion is coordinated by two bidentate ligands, one monodentate ligand and three water mol­ecules in the form of a distorted polyhedron with a coordination number of eight. Solvate water mol­ecules located between adjacent layers participate as donors and acceptors in a system of hydrogen bonds in which coordinating water mol­ecules also act as donors and non-coordinating carboxyl­ate O atoms act as acceptors.

## Related literature

For the crystal structures of Ca^II^ complexes with pyrazine-2,6-dicarboxyl­ate and water ligands, see: Starosta *et al.* (2003 ▶, 2004 ▶). The crystal structure of pyrimidine-4,6-dicarb­oxy­lic acid dihydrate was reported by Beobide *et al.* (2007 ▶).
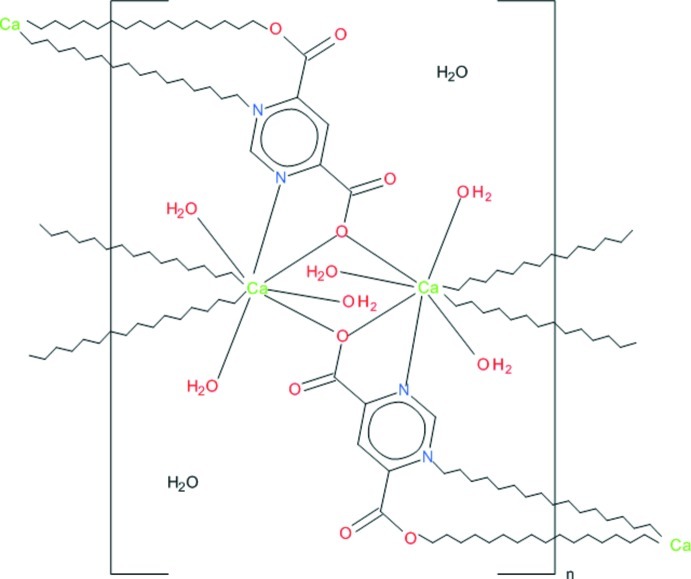


## Experimental

### 

#### Crystal data

[Ca_2_(C_6_H_2_N_2_O_4_)_2_(H_2_O)_6_]·2H_2_O
*M* *_r_* = 278.24
Monoclinic, 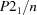

*a* = 7.7053 (15) Å
*b* = 11.432 (2) Å
*c* = 11.916 (2) Å
β = 92.16 (3)°
*V* = 1048.9 (4) Å^3^
*Z* = 4
Mo *K*α radiation
μ = 0.64 mm^−1^
*T* = 293 K
0.10 × 0.04 × 0.03 mm

#### Data collection

Kuma KM-4 four-circle diffractometer
Absorption correction: analytical (*CrysAlis RED*; Oxford Diffraction, 2008 ▶) *T*_min_ = 0.968, *T*_max_ = 0.982
3274 measured reflections
3065 independent reflections
1709 reflections with *I* > 2σ(*I*)
*R*_int_ = 0.049
3 standard reflections every 200 reflections intensity decay: 2.9%

#### Refinement

*R*[*F* ^2^ > 2σ(*F* ^2^)] = 0.033
*wR*(*F* ^2^) = 0.107
*S* = 1.01
3065 reflections
186 parameters
H atoms treated by a mixture of independent and constrained refinement
Δρ_max_ = 0.44 e Å^−3^
Δρ_min_ = −0.59 e Å^−3^

### 

Data collection: *KM-4 Software* (Kuma, 1996 ▶); cell refinement: *KM-4 Software*; data reduction: *DATAPROC* (Kuma, 2001 ▶); program(s) used to solve structure: *SHELXS97* (Sheldrick, 2008 ▶); program(s) used to refine structure: *SHELXL97* (Sheldrick, 2008 ▶); molecular graphics: *SHELXTL* (Sheldrick, 2008 ▶); software used to prepare material for publication: *SHELXTL*.

## Supplementary Material

Crystal structure: contains datablock(s) I, global. DOI: 10.1107/S160053681204617X/qm2088sup1.cif

Structure factors: contains datablock(s) I. DOI: 10.1107/S160053681204617X/qm2088Isup2.hkl

Additional supplementary materials:  crystallographic information; 3D view; checkCIF report

## Figures and Tables

**Table 1:** Selected bond lengths (Å)

Ca1—O5	2.398 (2)
Ca1—O7	2.420 (2)
Ca1—O3^i^	2.4361 (17)
Ca1—O1^ii^	2.4823 (16)
Ca1—O6	2.502 (2)
Ca1—O1	2.5061 (17)
Ca1—N1	2.563 (2)
Ca1—N3^i^	2.6133 (19)

Symmetry codes: (i) 
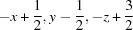
; (ii) 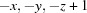.

**Table 2:** Hydrogen-bond geometry (Å, °)

*D*—H⋯*A*	*D*—H	H⋯*A*	*D*⋯*A*	*D*—H⋯*A*
O7—H72⋯O2^iii^	0.80 (6)	2.34 (6)	3.048 (3)	149 (6)
O6—H61⋯O2^ii^	0.85 (5)	1.88 (5)	2.698 (3)	163 (5)
O8—H81⋯O3^iv^	0.76 (4)	2.04 (4)	2.793 (3)	170 (4)
O8—H82⋯O6^v^	0.77 (5)	2.09 (5)	2.818 (3)	157 (5)
O5—H52⋯O8^vi^	0.87 (4)	1.94 (4)	2.798 (3)	169 (4)
O6—H62⋯O4^iii^	0.77 (4)	1.96 (4)	2.719 (2)	167 (4)
O7—H71⋯O4^vii^	0.79 (4)	2.16 (4)	2.902 (3)	158 (3)
O5—H51⋯O8^iv^	0.78 (4)	2.04 (4)	2.816 (3)	176 (4)

Symmetry codes: (ii) 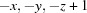; (iii) 
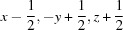
; (iv) 
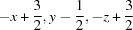
; (v) 
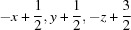
; (vi) 
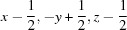
; (vii) 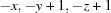.

**Fig. 1.**
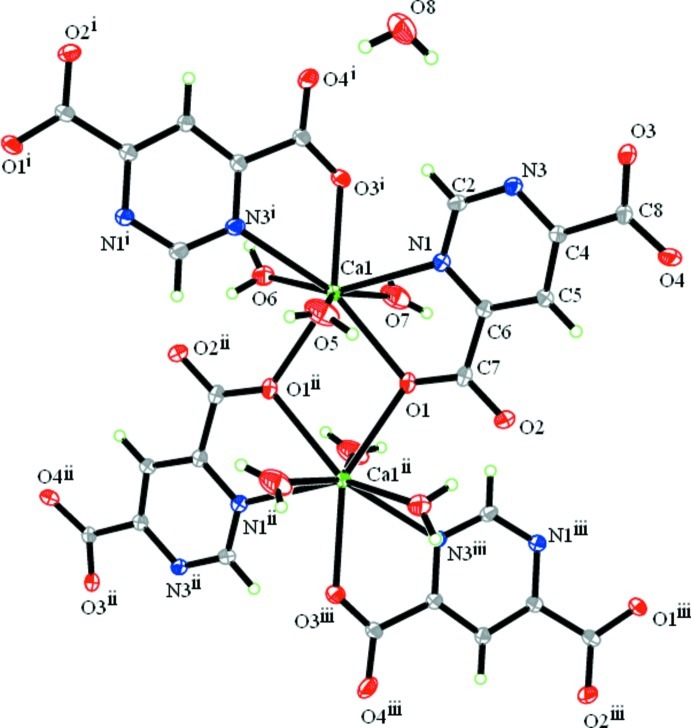
A fragment of the molecular layer showing a dimeric moiety with atom labelling scheme and 50% probability displacement ellipsoids. Symmetry code: i -x + 1/2, y - 1/2, -z + 3/2; ii -x, -y,-z + 1; iiix - 1/2, -y + 1/2, z + 1/2.

**Fig. 2.**
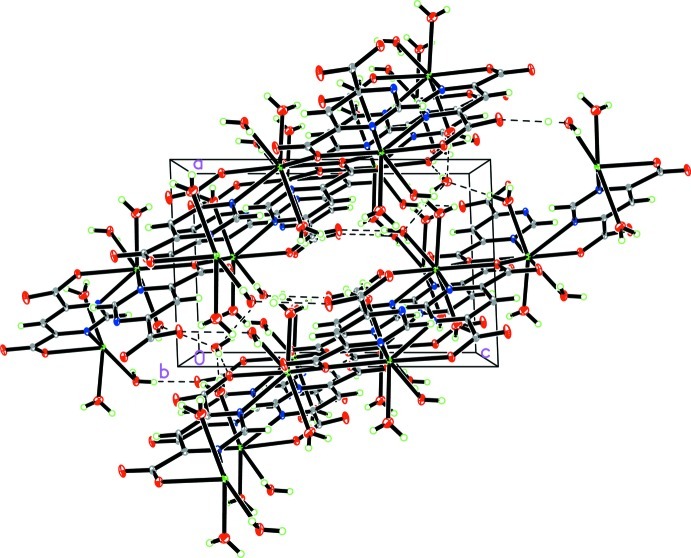
The alignment of molecular layers in the structure of a CaII complex with pyrimidine-4,6-carboxylate and water ligands viewed along [010].

## supplementary crystallographic information

### Comment

The asymmetric unit of the title compound contains one Ca^II^ ion, one fully deprotonated pyrimidine-4,6-diarboxylate ligand molecule, three water molecules coordinated to the metal ion and one solvation water molecule. The ligand molecule acts in µ_3_ mode and bridges Ca ^II^ ions using monodentate N and O atoms and another O atom which is bidentate. One of the O atoms in the carboxylate groups remains uncoordinated. Ca1 and Ca1^ii^ ions are related by an inversion centre and bridged by bidentate carboxylato O1 and O1^ii^ atoms to form a dimeric moiety (Fig.1). Due to the bridging action of the ligands the dimers are the nodes of a cross-linked two dimensional molecular network. The structure of the title compound can be thus visualized as built of molecular layers parallel to the crystal (101) plane (Fig.2). Solvation water molecules are located between adjacent layers. The Ca^II^ ion has a distorted eight coordinate geometry. The observed Ca—N and Ca—O bond distances are typical (Table 1). The pyrimidine ring is planar with an r.m.s. of 0.0079 (2) Å; carboxylate groups C7/O1/O2 and C8/O3/O4 make dihedral angles with the plane of 9.7 (1)° and 8.9 (1)°, respectively. Bond distances and bond angles within the pyrimidine ring do not differ from those reported for the parent acid (Beobide, *et al.*, 2007). In a system of hydrogen bonds, which is responsible for structure stability, solvation and coordinated water molecules act as donors and as acceptors and coordination inactive carboxylato O atoms act as acceptors (Table 2). Centro-symmetric dimeric units in which two Ca^II^ions are bridged by ligand bidentate carboxylato O atoms have been also observed in the structures of complexes with pyrazine-2,6-dicarboxylate and water ligands. In all, dimeric units bridged by pairs of coordinated aqua O atoms form "ladder" type molecular ribbons (Starosta, *et al.*, 2003, 2004).

### Experimental

An aqueous solution containing 1 mmol of calcium acetate hydrate and 1 mmol of pyrimidine-4,6-diarboxylic acid dihydrate was refluxed with constant stirring for 6 h. After cooling to room temperature, the solution was left to evaporate. Well formed single-crystal blocks appeared overnight at the bottom of the reaction pot. They were separated from the mother liquid, washed with cold water and dried in air.

### Refinement

Hydrogen atoms attached to water molecules were located in a difference map and refined isotropically, while two H atoms attached to pyrimidine C atoms were located at a calculated positions and treated as riding on the parent atoms with C—H=0.93 Å and

### Crystal data

**Table:**

[Ca_2_(C_6_H_2_N_2_O_4_)_2_(H_2_O)_6_]·2H_2_O	*F*(000) = 576
*M**_r_* = 278.24	*D*_x_ = 1.762 Mg m^−^^3^
Monoclinic, *P*2_1_/*n*	Mo *K*α radiation, λ = 0.71073 Å
Hall symbol: -P 2yn	Cell parameters from 25 reflections
*a* = 7.7053 (15) Å	θ = 6–15°
*b* = 11.432 (2) Å	µ = 0.64 mm^−^^1^
*c* = 11.916 (2) Å	*T* = 293 K
β = 92.16 (3)°	Blocks, colourless
*V* = 1048.9 (4) Å^3^	0.10 × 0.04 × 0.03 mm
*Z* = 4	

### Data collection

**Table:**

Kuma KM-4 four-circle diffractometer	1709 reflections with *I* > 2σ(*I*)
Radiation source: fine-focus sealed tube	*R*_int_ = 0.049
Graphite monochromator	θ_max_ = 30.1°, θ_min_ = 2.5°
profile data from ω/2θ scans	*h* = 0→10
Absorption correction: analytical (*CrysAlis RED*; Oxford Diffraction, 2008)	*k* = −16→0
*T*_min_ = 0.968, *T*_max_ = 0.982	*l* = −16→16
3274 measured reflections	3 standard reflections every 200 reflections
3065 independent reflections	intensity decay: 2.9%

### Refinement

**Table:**

Refinement on *F*^2^	Primary atom site location: structure-invariant direct methods
Least-squares matrix: full	Secondary atom site location: difference Fourier map
*R*[*F*^2^ > 2σ(*F*^2^)] = 0.033	Hydrogen site location: inferred from neighbouring sites
*wR*(*F*^2^) = 0.107	H atoms treated by a mixture of independent and constrained refinement
*S* = 1.01	*w* = 1/[σ^2^(*F*_o_^2^) + (0.0572*P*)^2^ + 0.0532*P*] where *P* = (*F*_o_^2^ + 2*F*_c_^2^)/3
3065 reflections	(Δ/σ)_max_ = 0.001
186 parameters	Δρ_max_ = 0.44 e Å^−^^3^
0 restraints	Δρ_min_ = −0.59 e Å^−^^3^

### Special details

**Table:**

Geometry. All e.s.d.'s (except the e.s.d. in the dihedral angle between two l.s. planes) are estimated using the full covariance matrix. The cell e.s.d.'s are taken into account individually in the estimation of e.s.d.'s in distances, angles and torsion angles; correlations between e.s.d.'s in cell parameters are only used when they are defined by crystal symmetry. An approximate (isotropic) treatment of cell e.s.d.'s is used for estimating e.s.d.'s involving l.s. planes.
Refinement. Refinement of *F*^2^ against ALL reflections. The weighted *R*-factor *wR* and goodness of fit *S* are based on *F*^2^, conventional *R*-factors *R* are based on *F*, with *F* set to zero for negative *F*^2^. The threshold expression of *F*^2^ > σ(*F*^2^) is used only for calculating *R*-factors(gt) *etc*. and is not relevant to the choice of reflections for refinement. *R*-factors based on *F*^2^ are statistically about twice as large as those based on *F*, and *R*- factors based on ALL data will be even larger.

### Fractional atomic coordinates and isotropic or equivalent isotropic displacement parameters (Å^2^)

**Table:**

	*x*	*y*	*z*	*U*_iso_*/*U*_eq_	
Ca1	0.03092 (6)	0.05117 (4)	0.65920 (3)	0.01409 (11)	
O1	0.0060 (2)	0.12495 (14)	0.46120 (13)	0.0195 (3)	
O2	0.0320 (3)	0.27178 (16)	0.33877 (13)	0.0261 (4)	
N1	0.1313 (3)	0.26241 (16)	0.63011 (14)	0.0163 (4)	
C6	0.1190 (3)	0.30679 (18)	0.52650 (17)	0.0148 (4)	
C5	0.1834 (3)	0.41685 (18)	0.50185 (18)	0.0174 (4)	
H5	0.1705	0.4487	0.4302	0.021*	
C7	0.0438 (3)	0.22887 (19)	0.43434 (18)	0.0165 (4)	
O4	0.3231 (3)	0.64500 (15)	0.48018 (14)	0.0313 (5)	
O7	−0.2529 (3)	0.14194 (19)	0.6459 (2)	0.0342 (5)	
O6	−0.1678 (3)	−0.08219 (17)	0.76038 (16)	0.0267 (4)	
N3	0.2839 (3)	0.43217 (16)	0.69222 (14)	0.0169 (4)	
O5	0.3228 (3)	0.0407 (2)	0.59679 (18)	0.0348 (5)	
C2	0.2132 (3)	0.32771 (19)	0.70832 (18)	0.0180 (4)	
H2	0.2216	0.2974	0.7807	0.022*	
C4	0.2679 (3)	0.47703 (19)	0.58880 (17)	0.0152 (4)	
C8	0.3517 (3)	0.59611 (18)	0.57191 (18)	0.0170 (4)	
O8	0.9331 (3)	0.35726 (19)	0.89833 (19)	0.0303 (4)	
H51	0.390 (5)	−0.010 (4)	0.601 (3)	0.047 (11)*	
O3	0.4436 (2)	0.63449 (14)	0.65273 (13)	0.0199 (3)	
H71	−0.285 (4)	0.187 (3)	0.600 (3)	0.031 (9)*	
H62	−0.174 (5)	−0.090 (3)	0.824 (3)	0.038 (10)*	
H52	0.358 (5)	0.081 (4)	0.540 (3)	0.055 (12)*	
H82	0.882 (6)	0.384 (5)	0.847 (4)	0.076 (16)*	
H81	0.957 (5)	0.293 (3)	0.888 (3)	0.036 (9)*	
H61	−0.120 (6)	−0.146 (4)	0.743 (4)	0.081 (16)*	
H72	−0.324 (8)	0.138 (5)	0.693 (5)	0.10 (2)*	

### Atomic displacement parameters (Å^2^)

**Table:**

	*U*^11^	*U*^22^	*U*^33^	*U*^12^	*U*^13^	*U*^23^
Ca1	0.0198 (2)	0.01036 (17)	0.01203 (17)	−0.00052 (18)	−0.00070 (13)	−0.00012 (16)
O1	0.0290 (9)	0.0126 (7)	0.0168 (7)	−0.0043 (6)	−0.0002 (6)	−0.0028 (6)
O2	0.0448 (10)	0.0193 (8)	0.0135 (7)	−0.0052 (8)	−0.0076 (7)	0.0006 (6)
N1	0.0223 (9)	0.0135 (8)	0.0131 (8)	−0.0017 (7)	−0.0013 (7)	−0.0006 (7)
C6	0.0180 (10)	0.0128 (9)	0.0135 (9)	−0.0005 (8)	−0.0010 (8)	−0.0012 (7)
C5	0.0266 (11)	0.0124 (9)	0.0130 (8)	−0.0024 (8)	−0.0019 (8)	0.0011 (7)
C7	0.0205 (10)	0.0135 (9)	0.0155 (9)	−0.0016 (8)	−0.0009 (8)	−0.0038 (8)
O4	0.0595 (14)	0.0183 (8)	0.0154 (8)	−0.0108 (9)	−0.0070 (8)	0.0064 (6)
O7	0.0323 (11)	0.0327 (11)	0.0378 (11)	0.0116 (9)	0.0063 (9)	0.0124 (9)
O6	0.0394 (11)	0.0236 (9)	0.0172 (8)	−0.0052 (8)	0.0030 (8)	0.0011 (7)
N3	0.0241 (9)	0.0149 (9)	0.0117 (7)	−0.0054 (7)	0.0003 (7)	0.0005 (6)
O5	0.0301 (10)	0.0360 (12)	0.0389 (11)	0.0114 (9)	0.0109 (8)	0.0158 (9)
C2	0.0264 (12)	0.0164 (10)	0.0112 (8)	−0.0030 (9)	−0.0013 (8)	0.0013 (8)
C4	0.0206 (10)	0.0123 (9)	0.0125 (9)	−0.0006 (8)	0.0001 (8)	−0.0001 (7)
C8	0.0257 (12)	0.0116 (9)	0.0136 (9)	−0.0029 (9)	0.0013 (8)	−0.0012 (7)
O8	0.0296 (10)	0.0219 (10)	0.0392 (11)	0.0042 (8)	0.0005 (8)	−0.0076 (8)
O3	0.0298 (9)	0.0132 (7)	0.0164 (7)	−0.0064 (7)	−0.0026 (6)	−0.0015 (6)

### Geometric parameters (Å, º)

**Table:**

Ca1—O5	2.398 (2)	C5—H5	0.9300
Ca1—O7	2.420 (2)	O4—C8	1.240 (3)
Ca1—O3^i^	2.4361 (17)	O7—H71	0.79 (4)
Ca1—O1^ii^	2.4823 (16)	O7—H72	0.80 (6)
Ca1—O6	2.502 (2)	O6—H62	0.77 (4)
Ca1—O1	2.5061 (17)	O6—H61	0.85 (5)
Ca1—N1	2.563 (2)	N3—C2	1.330 (3)
Ca1—N3^i^	2.6133 (19)	N3—C4	1.336 (3)
Ca1—Ca1^ii^	3.9817 (11)	N3—Ca1^iii^	2.6133 (19)
Ca1—H61	2.74 (5)	O5—H51	0.78 (4)
O1—C7	1.267 (3)	O5—H52	0.87 (4)
O1—Ca1^ii^	2.4823 (16)	C2—H2	0.9300
O2—C7	1.240 (3)	C4—C8	1.523 (3)
N1—C2	1.334 (3)	C8—O3	1.253 (3)
N1—C6	1.335 (3)	O8—H82	0.77 (5)
C6—C5	1.388 (3)	O8—H81	0.76 (4)
C6—C7	1.512 (3)	O3—Ca1^iii^	2.4361 (17)
C5—C4	1.385 (3)		
			
O5—Ca1—O7	148.95 (7)	N1—Ca1—H61	163.4 (11)
O5—Ca1—O3^i^	105.11 (8)	N3^i^—Ca1—H61	63.5 (11)
O7—Ca1—O3^i^	86.21 (7)	Ca1^ii^—Ca1—H61	93.9 (11)
O5—Ca1—O1^ii^	82.45 (7)	C7—O1—Ca1^ii^	129.56 (14)
O7—Ca1—O1^ii^	103.09 (7)	C7—O1—Ca1	122.89 (13)
O3^i^—Ca1—O1^ii^	148.46 (6)	Ca1^ii^—O1—Ca1	105.91 (6)
O5—Ca1—O6	135.80 (7)	C2—N1—C6	116.67 (19)
O7—Ca1—O6	74.03 (7)	C2—N1—Ca1	124.75 (14)
O3^i^—Ca1—O6	79.90 (6)	C6—N1—Ca1	118.15 (14)
O1^ii^—Ca1—O6	74.06 (6)	N1—C6—C5	121.77 (19)
O5—Ca1—O1	76.37 (7)	N1—C6—C7	117.37 (19)
O7—Ca1—O1	75.95 (7)	C5—C6—C7	120.70 (19)
O3^i^—Ca1—O1	137.32 (5)	C4—C5—C6	116.97 (19)
O1^ii^—Ca1—O1	74.09 (6)	C4—C5—H5	121.5
O6—Ca1—O1	129.21 (6)	C6—C5—H5	121.5
O5—Ca1—N1	73.37 (7)	O2—C7—O1	126.4 (2)
O7—Ca1—N1	82.17 (7)	O2—C7—C6	116.52 (19)
O3^i^—Ca1—N1	75.03 (6)	O1—C7—C6	117.02 (19)
O1^ii^—Ca1—N1	135.61 (6)	Ca1—O7—H71	126 (2)
O6—Ca1—N1	146.35 (7)	Ca1—O7—H72	125 (4)
O1—Ca1—N1	64.43 (5)	H71—O7—H72	109 (5)
O5—Ca1—N3^i^	71.93 (7)	Ca1—O6—H62	127 (3)
O7—Ca1—N3^i^	137.41 (7)	Ca1—O6—H61	97 (3)
O3^i^—Ca1—N3^i^	63.72 (6)	H62—O6—H61	101 (4)
O1^ii^—Ca1—N3^i^	90.98 (6)	C2—N3—C4	116.97 (18)
O6—Ca1—N3^i^	71.62 (7)	C2—N3—Ca1^iii^	125.87 (14)
O1—Ca1—N3^i^	146.45 (6)	C4—N3—Ca1^iii^	117.02 (14)
N1—Ca1—N3^i^	114.97 (6)	Ca1—O5—H51	131 (3)
O5—Ca1—Ca1^ii^	76.69 (6)	Ca1—O5—H52	122 (3)
O7—Ca1—Ca1^ii^	89.33 (6)	H51—O5—H52	103 (4)
O3^i^—Ca1—Ca1^ii^	173.73 (4)	N3—C2—N1	126.0 (2)
O1^ii^—Ca1—Ca1^ii^	37.25 (4)	N3—C2—H2	117.0
O6—Ca1—Ca1^ii^	103.10 (5)	N1—C2—H2	117.0
O1—Ca1—Ca1^ii^	36.84 (4)	N3—C4—C5	121.6 (2)
N1—Ca1—Ca1^ii^	100.01 (4)	N3—C4—C8	116.09 (18)
N3^i^—Ca1—Ca1^ii^	122.36 (5)	C5—C4—C8	122.31 (18)
O5—Ca1—H61	119.2 (10)	O4—C8—O3	126.6 (2)
O7—Ca1—H61	88.9 (10)	O4—C8—C4	117.21 (19)
O3^i^—Ca1—H61	90.5 (11)	O3—C8—C4	116.20 (18)
O1^ii^—Ca1—H61	60.1 (11)	H82—O8—H81	112 (4)
O6—Ca1—H61	17.9 (10)	C8—O3—Ca1^iii^	126.43 (14)
O1—Ca1—H61	126.9 (11)		

Symmetry codes: (i) −*x*+1/2, *y*−1/2, −*z*+3/2; (ii) −*x*, −*y*, −*z*+1; (iii) −*x*+1/2, *y*+1/2, −*z*+3/2.

### Hydrogen-bond geometry (Å, º)

**Table:**

*D*—H···*A*	*D*—H	H···*A*	*D*···*A*	*D*—H···*A*
O7—H72···O2^iv^	0.80 (6)	2.34 (6)	3.048 (3)	149 (6)
O6—H61···O2^ii^	0.85 (5)	1.88 (5)	2.698 (3)	163 (5)
O8—H81···O3^v^	0.76 (4)	2.04 (4)	2.793 (3)	170 (4)
O8—H82···O6^iii^	0.77 (5)	2.09 (5)	2.818 (3)	157 (5)
O5—H52···O8^vi^	0.87 (4)	1.94 (4)	2.798 (3)	169 (4)
O6—H62···O4^iv^	0.77 (4)	1.96 (4)	2.719 (2)	167 (4)
O7—H71···O4^vii^	0.79 (4)	2.16 (4)	2.902 (3)	158 (3)
O5—H51···O8^v^	0.78 (4)	2.04 (4)	2.816 (3)	176 (4)

Symmetry codes: (ii) −*x*, −*y*, −*z*+1; (iii) −*x*+1/2, *y*+1/2, −*z*+3/2; (iv) *x*−1/2, −*y*+1/2, *z*+1/2; (v) −*x*+3/2, *y*−1/2, −*z*+3/2; (vi) *x*−1/2, −*y*+1/2, *z*−1/2; (vii) −*x*, −*y*+1, −*z*+1.
